# Reductive Cytochrome P450 Reactions and Their Potential Role in Bioremediation

**DOI:** 10.3389/fmicb.2021.649273

**Published:** 2021-04-15

**Authors:** James B. Y. H. Behrendorff

**Affiliations:** ^1^Centre for Agriculture and the Bioeconomy, Queensland University of Technology, Brisbane, QLD, Australia; ^2^Commonwealth Scientific and Industrial Research Organisation (CSIRO) Synthetic Biology Future Science Platform, Canberra, ACT, Australia

**Keywords:** synthetic biology, denitration, dehalogenation, bioremediation, cytochrome P450

## Abstract

Cytochrome P450 enzymes, or P450s, are haem monooxygenases renowned for their ability to insert one atom from molecular oxygen into an exceptionally broad range of substrates while reducing the other atom to water. However, some substrates including many organohalide and nitro compounds present little or no opportunity for oxidation. Under hypoxic conditions P450s can perform reductive reactions, contributing electrons to drive reductive elimination reactions. P450s can catalyse dehalogenation and denitration of a range of environmentally persistent pollutants including halogenated hydrocarbons and nitroamine explosives. P450-mediated reductive dehalogenations were first discovered in the context of human pharmacology but have since been observed in a variety of organisms. Additionally, P450-mediated reductive denitration of synthetic explosives has been discovered in bacteria that inhabit contaminated soils. This review will examine the distribution of P450-mediated reductive dehalogenations and denitrations in nature and discuss synthetic biology approaches to developing P450-based reagents for bioremediation.

## Highlights

- Complete bioremediation of many organohalide and nitro compounds requires a combination of reductive and oxidative reactions, but many reductive dehalogenases are highly oxygen sensitive. This makes their use in bioremediation more challenging.
- Cytochrome P450 enzymes are aerotolerant enzymes renowned for their oxidative monooxygenation reactions, but they can also perform reductive elimination reactions under certain conditions.
- Reduction of pollutants by cytochrome P450 enzymes can drive dehalogenation and denitration reactions that could be useful for bioremediation.

## Introduction

The late nineteenth and twentieth centuries saw explosive growth in the production and dissemination of halogenated and nitrated abiotic chemicals such as solvents, pesticides, and fire suppressants. Many of these chemicals are highly toxic and some persist in the environment due to the presence of halogen groups that do not spontaneously oxidise at meaningful rates. These include poly- and perfluoroalkyl substance (PFAS) “forever chemicals” that accumulate and persist in environments and food chains, but also other halogenated and nitrated compounds the degrade extremely slowly. For example, abiotic hydrolysis of carbon tetrachloride in groundwater is so slow that the compound is estimated to have a half-life of 630 years (Amonette et al., 2012).

Oxidative or hydrolytic enzymes that dehalogenate organic compounds (organohalides) have been identified in a variety of organisms either as components of respiratory processes or in catabolic pathways, but some organohalides such as highly halogenated alkanes are highly resistant to oxidation (Fetzner, 1998).

Organohalide-respiring bacteria can metabolise some perhalogenated alkanes by coupling the reductive elimination of a carbon-halide bond to energy conservation, ultimately using the change in Gibb’s free energy from reductive dehalogenation to drive ATP synthesis (Fincker and Spormann, 2017). But these organisms are slow-growing and their dehalogenase enzymes are highly oxygen sensitive, which limits their ecological distribution. Also in many cases these organisms only catalyse partial metabolism of anthropogenic organohalides. These factors limit their utility for interventionist bioremediation efforts.

A further challenge is that contaminated environments usually contain a mixture of related pollutant compounds. Effective bioremediation of halogenated compounds will likely require a combination of reductive and oxidative reactions, and a diversity of enzymes that can handle mixtures of compounds. Rather than relying solely on native biological systems, scientists are increasingly turning to synthetic biology to combine and adapt the most useful biological parts from nature into bespoke organisms for bioremediation (Rylott and Bruce, 2020).

Cytochrome P450 enzymes (P450s) are extremely versatile redox enzymes that may play important roles in future synthetic bioremediation agents. P450s are a family of haem-thiolate enzymes renowned for their general monooxygenase function where they insert one atom from molecular oxygen into a substrate, usually increasing the solubility of relatively hydrophobic compounds, while reducing the other oxygen atom to water. P450 ligand access channels can accept poorly soluble substrates (Urban et al., 2018), and the volume and malleability of P450 active sites varies widely with substrates ranging in molecular weight from methane (Zilly et al., 2011) (16 g/mol) to erythromycin (Kalsotra et al., 2004) (734 g/mol). Their ability to oxygenate an extraordinary range of compounds including unactivated carbon centres via high-valent iron redox chemistry has earned P450s the nickname “nature’s blowtorch”(Guengerich, 2009). They have attracted interest for their potential use in bioremediation, where many substrates are recalcitrant due to their high hydrophobicity and the presence of difficult-to-metabolise nitro or halogen groups (Kellner et al., 1997; Behrendorff and Gillam, 2016).

Many P450s have been identified that catalyse the initial oxidation step in the catabolism of persistent organic pollutants such as polycyclic aromatic hydrocarbons (PAHs). Native (England et al., 1998; Syed et al., 2010) and engineered (Sideri et al., 2013; Syed et al., 2013) P450s have been identified that can hydroxylate hydrophobic high-molecular weight PAHs such as naphthalene (C_1__0_H_8_), phenanthrene (C_1__4_H_10_), pyrene (C_1__6_H_10_), and chrysene (C_1__8_H_12_). Beyond oxidation of hydrocarbons, several P450s can perform oxidative dehalogenations. For example, P450_*CAM*_, a camphor-oxidising P450 from *Pseudomonas putida*, can oxidise 1,2-dichloropropane to chloroacetone (Lefever and Wackett, 1994) and engineered mutants of this enzyme can hydroxylate polychlorinated benzenes (Jones et al., 2001).

In addition to oxidative metabolism of organohalides (extensively reviewed elsewhere; Isin and Guengerich, 2007; Guengerich, 2009; Guengerich and Munro, 2013), P450s can also catalyse reductive halide elimination reactions when their usual cosubstrate, molecular oxygen, is absent. As of 2018, over 300,000 cytochrome P450 sequences had been identified in sequence databases (Nelson, 2018) and although there are few comprehensive studies of reductive P450 reactions it seems likely that known P450-mediated reductive dehalogenation reactions are only a small fraction of what is possible.

Cytochrome P450 enzymes may be a useful resource for developing biocatalysts that perform reductive elimination reactions for bioremediation of pollutants. Because P450s are naturally aerotolerant they can be deployed with greater flexibility than other reductive dehalogenases that are usually highly sensitive to oxygen. Furthermore there is a strong track record of engineering P450s to enhance functional properties like reaction selectivity (Hunter et al., 2011; Jung et al., 2011), expression yield in heterologous hosts (Barnes et al., 1991; Behrendorff et al., 2012), and thermostability and solvent tolerance (Salazar et al., 2003; Gumulya et al., 2018)—all important factors for developing efficient synthetic biocatalysts.

This review specifically focuses on known P450-mediated reductive dehalogenation and denitration reactions and discusses the potential for exploiting reductive P450 reactions in bioremediation.

## The Cytochrome P450 Catalytic Cycle

Cytochrome P450 enzymes (P450s) are found in organisms across the tree of life, and as such they occupy myriad biological niches. Bacterial P450s are often involved in oxidation of environmental chemicals to facilitate their catabolism, whereas many plant P450s catalyse key steps in biosynthesis of secondary metabolites or oxidation of xenobiotics such as herbicides (Siminszky, 2006; Mizutani and Ohta, 2010). Mammalian P450s from liver microsomes have exceptional substrate promiscuity and are among the best studied P450s due to their importance in drug metabolism. The structure and malleability of the active site can vary dramatically between different P450 enzymes, and many mammalian P450s can accept a wide range of substrates and differ in their product spectra (Isin and Guengerich, 2007). Collectively P450s can perform carbon or heteroatom hydroxylations, epoxidations and dealkylations on a staggering array of substrates (Isin and Guengerich, 2007). P450s have also been discovered that perform less common reactions including nitrations (Guengerich and Munro, 2013) and C-C bond formation or cleavage (Guengerich and Yoshimoto, 2018).

**FIGURE 1:**
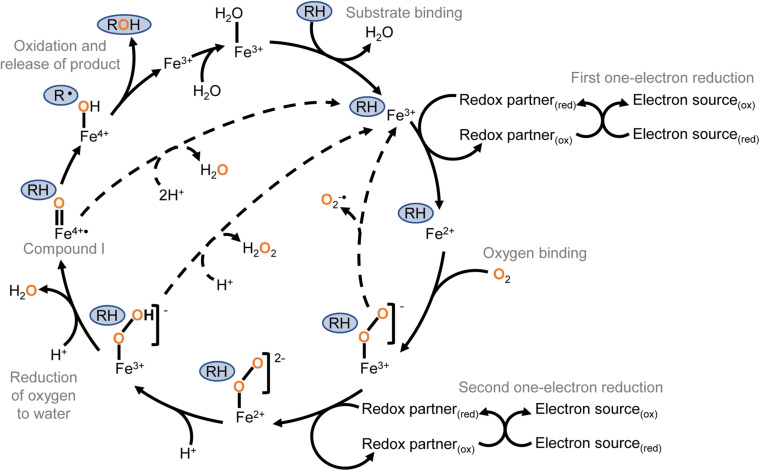
The conventional cytochrome P450 catalytic cycle. Key steps are labelled with grey text. The substrate is marked in a blue oval, and oxygen atoms originating from molecular oxygen are shown in orange. The conventional cycle is shown with solid arrows, and uncoupling routes with dashed arrows. The cycle begins with the enzyme in the “resting state” where a water ligand occupies the active site and the haem iron is in the ferric state. Substrate binding displaces the water molecule and is followed by a one-electron reduction of the haem iron from Fe^3+^ to Fe^2+^, creating the opportunity for molecular oxygen to bind the ferrous haem. A second one-electron reduction creates a ferric-peroxo intermediate that is quickly protonated to a ferric-hydroperoxo species. A second protonation results in the release of water and formation of the highly reactive ferryl-oxo porphyrin radical known as “compound I.” Compound I abstracts a hydrogen atom from the substrate, briefly forming a ferryl hydroxide that recombines with the highly reactive substrate radical that resulted from the proton abstraction. The net result of this reaction cascade is monooxygenation of the substrate to form the product and return the haem iron to the ferric state. The P450 catalytic cycle can uncouple (dashed arrows) after either of the one-electron reduction steps, resulting in the release of reduced oxygen as superoxide or hydrogen peroxide. The ferryl hydroxide can also be protonated to cause uncoupling and the release of water without metabolism of the substrate.

The conventional P450 catalytic cycle (Figure 1) begins with the enzyme in a “resting state” where a water ligand occupies the active site and the haem iron is in the ferric (Fe^3+^) state. Substrate binding displaces the water molecule and is followed by a one-electron reduction of the haem iron from Fe^3+^ to Fe^2+^, creating the opportunity for molecular oxygen to bind the ferrous haem. Some P450s have been observed in the one-electron-reduced state prior to substrate binding (Johnston et al., 2011). A second one-electron reduction creates a ferric-peroxo intermediate that is quickly protonated to a ferric-hydroperoxo species and then protonated again resulting in the release of water and formation of the highly reactive ferryl-oxo porphyrin radical known as “compound I.” Compound I abstracts a hydrogen atom from the substrate, briefly forming a ferryl hydroxide that recombines with the highly reactive substrate radical that resulted from the proton abstraction. The net result of this cascade is insertion of oxygen to form the reaction product and returning the haem iron to the ferric state (Rittle and Green, 2010).

The electrons required to drive the P450 catalytic cycle can come from a variety of sources. The redox midpoint potentials of ferrous P450 enzymes in the presence or absence of substrate are generally reported to be in the range of −300 to −225 mV, and in eukaryotic systems the first electron usually comes from oxidation of NAD(P)H by a diflavin reductase partner protein (cytochrome P450 reductase, CPR) which then transfers the electron to the P450. Similar systems have been identified in bacteria where the P450 is reduced either directly by a diflavin reductase, or by a flavodoxin or ferredoxin protein that serves as an intermediary between the P450 and a flavodoxin/ferredoxin reductase (Li et al., 2020). The ultimate source of reducing power does not have to be NAD(P)H, so long as a mechanism exists to transfer electrons to the P450 haem iron. This flexibility has facilitated development of synthetic biological systems where P450s can be reduced by the photosynthetic electron transport chain via various ferredoxin or flavodoxin intermediates (Jensen et al., 2011; Mellor et al., 2019). P450 activity can also be supported by a number of artificial mechanisms including direct provision of electrons via an electrode (Fleming et al., 2003; Fantuzzi et al., 2004), chemical reductants including sodium dithionite, titanium (III) citrate (Li and Wackett, 1993), reactive oxygen surrogates that bypass the first reductive steps of the reaction cycle (Strohmaier et al., 2020), or even from electron donors such as EDTA or triethanolamine in the presence of light and photosensitisers (such as proflavin or the fluorescein dye Eosin Y) (Park et al., 2015).

Although the net result of a typical P450 catalytic cycle is monooxygenation of a substrate, the haem iron itself is an oxygen reductase. The P450 catalytic cycle can uncouple after either of the one-electron reduction steps to release reduced oxygen in the form of superoxide or hydrogen peroxide. In the absence of oxygen, other compounds can be reduced by the catalytic haem including halogenated and nitrated pollutants.

## Understanding Reductive Cytochrome P450 Reactions

Shortly after the discovery of cytochrome P450 enzymes (Omura and Sato, 1962) there were hints that they could perform reductive reactions such as reduction of *p*-nitrobenzoate to *p*-aminobenzoate (Gillette et al., 1968). P450-mediated reductive dehalogenation reactions were also identified soon afterward. Metabolism of halothane, a halogenated anaesthetic drug, provided an early case study. Halothane was first synthesised in 1951 (Huang et al., 2017), meaning that its reductive dehalogenation is not an evolutionary adaptation of P450s but rather an illustrative example of their general catalytic capabilities. P450s metabolise halothane (CF_3_CHClBr) either in the presence or absence of oxygen (Van Dyke and Gandolf, 1976) and the mixture of reduced and oxidised dehalogenated products depends on the oxygen concentration (Nastainczyk et al., 1978). These initial studies of halothane metabolism predated recombinant expression of P450s in heterologous hosts, and it was not until the 1990s that reductive and oxidative metabolism of halothane was assigned to specific enzyme isoforms.

**FIGURE 2:**
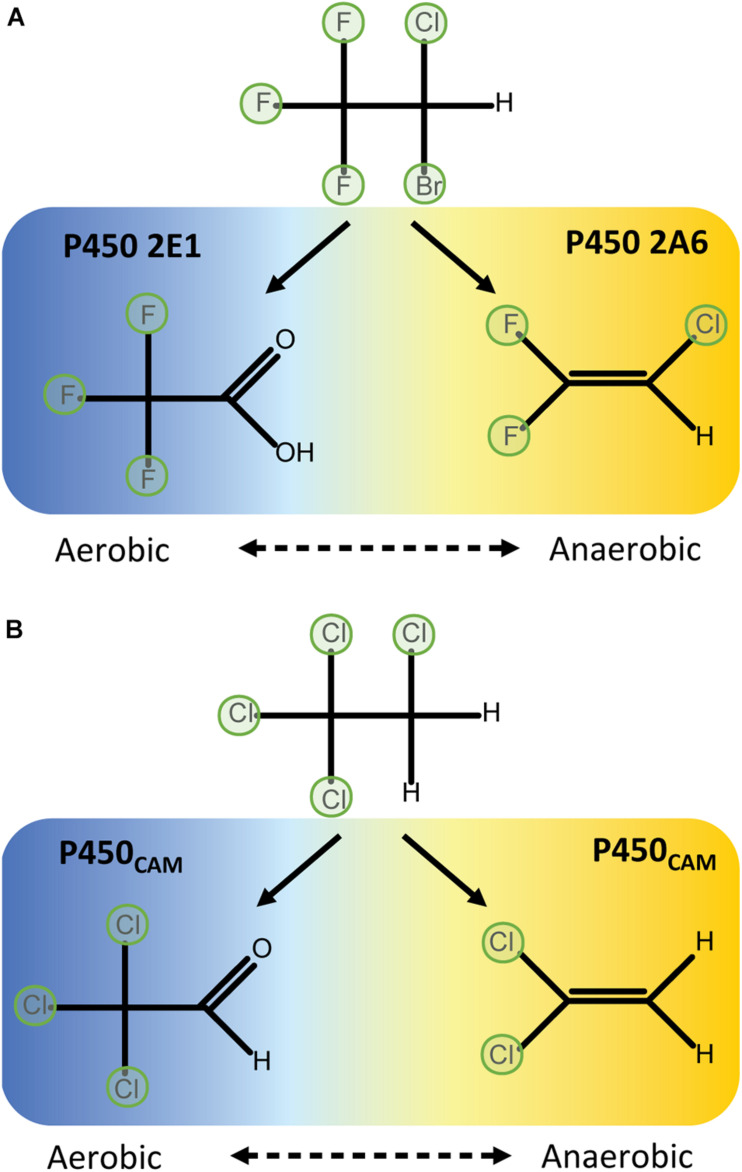
Aerobic oxidative dehalogenation and anaerobic reductive dehalogenation by P450 enzymes. **(A)** In human liver microsomes, halothane is metabolised to difluoro-chloroethylene by P450 2A6 in hypoxic conditions or trifluoroacetic acid by P450 2E1 in aerobic conditions (Spracklin et al., 1996). **(B)** P450_*C*__*AM*_ differentially metabolises 1,1,1,2-tetrachloroethane to either dichloroethylene in anaerobic conditions or trichloroacetaldehyde in aerobic conditions (Logan et al., 1993).

Reductive dehalogenation of halothane requires oxygen concentrations of less than 5% (Nastainczyk et al., 1978), which is within range of the conditions in the centrilobular regions of the liver (Lind et al., 1986). In oxidative conditions halothane is primarily metabolised to trifluoroacetic acid by P450 2E1. In oxygen-limited conditions P450s 2A6 and 3A4 produce the reduced products chloro-difluoroethylene and chloro-trifluoroethane (Spracklin et al., 1996) (Figure 2A). P450 2A6 also releases a radical intermediate that can initiate lipid peroxidation (Minoda and Kharasch, 2001). Although P450 2E1 appears to primarily metabolise halothane via the oxidative route, it can reduce other substrates at low oxygen tension including carbon tetrachloride (Koop, 1992). Collectively, these data indicate reductive metabolism is a general feature of P450s but that active site conformation and the orientation and redox properties of the substrate relative to the haem are important factors in determining whether reductive dehalogenation will occur.

Reductive dehalogenation and denitration reactions are initiated when an electron is transferred from the ferrous haem directly to the substrate, resulting in a substrate radical. After this point there are several possible outcomes, with the product distribution probably depending on the active site dynamics of the P450 and substrate involved. In the example of reductive halothane metabolism, the substrate is first reduced by a single electron from the ferrous haem. This results in bromide elimination and the formation of a [CF_3_CHCl]^•^ radical that can be protonated to form chlorotrifluoroethane or further reduced to yield chlorodifluoroethylene and inorganic fluoride (Minoda and Kharasch, 2001). In sum, reductive P450 reactions depend on a supply of reducing equivalents, reduction of the haem iron, and active site dynamics that position the substrate sufficiently close to the reduced haem.

## Distribution of Known Reductive P450 Reactions in Nature

Relatively few reductive P450 reactions have been discovered to date but those reactions that have been identified can often be performed by more than one P450 isoform and some common features have begun to emerge. The structure and malleability of the active site likely play important roles in determining whether collisions between the reduced haem and different substrates are possible. Examples of known P450-catalyzed reductive dehalogenation and denitration reactions are discussed below and summarised in Table 1.

**TABLE 1:** Summary of known P450-mediated reductive dehalogenation and denitration reactions.

**Substrate**	**Product**	**Notes**
Trichloronitromethane CCl_3_NO_2_	Nitromethane CH_3_NO_2_	P450_*C*__*AM*_ (*Pseudomonas putida*), *in vitro* assays with dithionite as reductant (Castro et al., 1985).
Bromotrichloromethane BrCCl_3_	Chloroform CHCl_3_	P450_*C*__*AM*_ (*Pseudomonas putida*), *in vitro* assays with dithionite (Castro et al., 1985) or titanium (III) citrate (Li and Wackett, 1993) as reductant.
Dibromodichloromethane Br_2_CCl_2_	Bromodichloromethane BrHCCl_2_	P450_*C*__*AM*_ (*Pseudomonas putida*), *in vitro* assays with titanium (III) citrate as reductant (Li and Wackett, 1993).
Bromodichloromethane BrHCCl_2_	Dichloromethane H_2_CCl_2_	P450_*C*__*AM*_ (*Pseudomonas putida*), *in vitro* assays with titanium (III) citrate as reductant (Li and Wackett, 1993).
Carbon tetrachloride CCl_4_	Chloroform CHCl_3_	P450_*C*__*AM*_ (*Pseudomonas putida*), *in vitro* assays dithionite (Castro et al., 1985) or titanium (III) citrate (Li and Wackett, 1993) as reductant.
	Trichloromethyl radical ^•^CCl_3_	P450 2E1 (*Homo sapiens*), *in vitro* assays of human liver microsomes and recombinant enzymes in insect cell microsomes with NADPH as reductant (Zangar et al., 2000). The radical product has multiple potential fates including enzyme inactivation.
	Methane CH_4_	P450 119 (*Sulfolobus solfataricus*), electrocatalysis using a thermostable enzyme immobilised on an electrode (Blair et al., 2004).
Hexachloroethane C_2_Cl_6_	Tetrachloroethylene C_2_Cl_4_	P450_*C*__*AM*_ (*Pseudomonas putida*) (Li and Wackett, 1993; Logan et al., 1993; Walsh et al., 2000) *In vitro* assays with titanium (III) citrate (Li and Wackett, 1993) or the putidaredoxin/putidaredoxin system and NADH as reductant (Walsh et al., 2000). *In vivo* assays in *Pseudomonas putida* (Logan et al., 1993) or with P450 1A2 (*Rattus norvegicus*) (Yanagita et al., 1997) recombinant P450 1A2 expressed in *S. cerevisiae*.
Pentachloroethane C_2_HCl_5_	Trichloroethylene C_2_HCl_3_	P450_*C*__*AM*_ (*Pseudomonas putida*) (Li and Wackett, 1993; Logan et al., 1993) P450 1A2 (*Rattus norvegicus*) (Yanagita et al., 1997) *In vitro* assays with titanium (III) citrate as reductant (Li and Wackett, 1993) or *in vivo* assays in *Pseudomonas putida* (Logan et al., 1993) or with recombinant P450 1A2 expressed in *S. cerevisiae* (Yanagita et al., 1997).
1,1,1,2-tetrachloroethane C_2_H_2_Cl_4_	1,1-dichloroethylene C_2_H_2_Cl_2_	P450_*C*__*AM*_ (*Pseudomonas putida*) *In vitro* assays with titanium (III) citrate as reductant (Li and Wackett, 1993) or *in vivo* assays in *Pseudomonas putida* (Logan et al., 1993).
Trifluorotrichloroethane F_3_C_2_Cl_3_	Tri- and di-fluorodichloroethane (F_3_C_2_HCl_2_, F_2_C_2_Cl_2_)	P450_*C*__*AM*_ (*Pseudomonas putida*) *In vitro* assays with titanium (III) citrate as reductant (Li and Wackett, 1993).
Halothane F_3_C_2_HClBr	Trifluorochloroethane F_3_C_2_H_2_Cl	P450_*C*__*AM*_ (*Pseudomonas putida*) *In vitro* assays with titanium (III) citrate as reductant (Li and Wackett, 1993).
	Tri- and di-fluorochloroethane (F_3_C_2_HCl_2_, F_2_C_2_Cl_2_)	P450s 2A6 and 3A4 (*Homo sapiens*) *In vitro* assays with human liver microsomes and NADPH as reductant (Spracklin et al., 1996).
Trichlorofluoromethane FCCl_3_	Carbon monoxide CO	P450_*C*__*AM*_ (*Pseudomonas putida*) *In vitro* assays with titanium (III) citrate as reductant (Li and Wackett, 1993). Carbon monoxide is proposed to form via dichlorofluoromethyl (FCCl_2_) and chlorofluorocarbene (FCCl) radical intermediates, where the chlorofluorocarbene radical is reduced to CO by water.
1,3,5-trinitro-1,3,5-triazinane (RDX, Royal Demolition Explosive) C_3_H_6_N_6_O_6_	Methylenedinitramine CH_4_N_4_O_4_	P450 XplA [*Rhodococcus rhodochrous* 11Y (Seth-Smith et al., 2002) and various other soil bacteria (Bhushan et al., 2003; Sabir et al., 2017)] P450 2B6 (*Oryctolagus cuniculus*) (Bhushan et al., 2003) *In vivo* assays in native and heterologous expression hosts (Seth-Smith et al., 2002; Bhushan et al., 2003). *In vitro* assays with XplB reductases and NADH as reductant (Sabir et al., 2017). Rabbit P450 2B6 *in vitro* assays with NAPDH:P450 reductase and NADPH as reductant (Bhushan et al., 2003)
Dichlorodiphenyl-trichloroethane (DDT) C_1__4_H_9_Cl_5_	Dichlorodiphenyl-dichloroethane (DDD) C_1__4_H_8_Cl_4_	P450s 2B6, 2C9, 2E1, 3A4 (*Rattus norvegicus*) (Kitamura et al., 2002) P450 6G1 (*Drosophila melanogaster*) (Joußen et al., 2008) *In vitro* assays using rat liver microsomes with NADPH as reductant (Kitamura et al., 2002), and *in vivo* assays with P450 6G1 expressed in tobacco cell culture (Joußen et al., 2008). The reaction can also proceed non-enzymatically [in the presence of reductants and heat-denatured enzyme (Kitamura et al., 2002) or haemoglobin (Kitamura et al., 1999)].

### Reductive Dechlorination of Halomethanes and Haloethanes

P450_*C*__*AM*_ is a soluble P450 from *Pseudomonas putida*. The physiological role of P450_*C*__*AM*_ is to hydroxylate camphor in the first step of camphor catabolism. P450_*C*__*AM*_ is reduced by a soluble 2Fe-2S ferredoxin protein (“putidaredoxin”) which in turn is reduced by NADH via the putidaredoxin reductase. Structure-function relationships in P450_*C*__*AM*_ have been studied extensively. P450_*C*__*AM*_ was the first P450 structure solved through X-ray crystallography (Poulos et al., 1987) and over 100 structures have been deposited in the RCSB Protein Data Bank^1^ showing P450_*C*__*AM*_ and engineered mutants in complex with various substrates, inhibitors, and redox partner proteins. As such, P450_*C*__*AM*_ is one of the best-studied non-human P450 enzymes and it was the first bacterial P450 enzyme where reductive dehalogenation reactions were conclusively identified (Castro et al., 1985).

**FIGURE 3:**
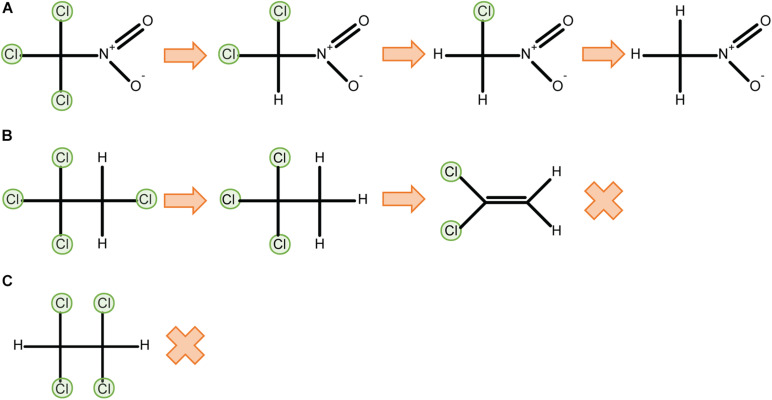
Physicochemical properties such as bond polarisation affect reductive dehalogenation. P450_*C*__*AM*_ can fully dechlorinate trichloronitromethane through reductive reactions **(A)**, but only partially dechlorinates 1,1,1,2-tetrachloroethane to 1,1-dichloroethylene **(B)**. 1,1,2,2-tetrachloroethane cannot be metabolised by P450_*C*__*AM*_ in reductive conditions **(C)** (Logan et al., 1993).

P450_*C*__*AM*_ catalyses two-electron reductions of a variety of halomethanes and haloethanes to yield dehalogenated products. That is, two sequential one-electron reductions occur to complete a reductive elimination reaction without releasing observable quantities of a one-electron-reduced radical intermediate. The extent to which perhalogenated compounds are dehalogenated depends on the physico-chemical properties of the substrate. For example, complete dehalogenation of trichloronitromethane (Cl_3_CNO_2_) to nitromethane via di- and mono-chloronitromethane intermediates (Castro et al., 1985) is likely aided by the electron-withdrawing properties of the nitro group. In contrast, tetrachloroethanes can only be partially dehalogenated by reductive reactions, possibly because of their reduced polarisation: P450_*C*__*AM*_ can dechlorinate 1,1,1,2-tetrachloroethane to dichloroethylene, but 1,1,2,2-tetrachloroethane is unreactive (Logan et al., 1993) (Figure 3). In aerobic conditions 1,1,1,2-tetrachloroethane is metabolised to 1,1,1-trichloroacetaldehyhde (Figure 2B).

Several active site mutants of P450_*C*__*AM*_ have been characterised. Using hexachloroethane and pentachloroethane as model substrates, *in vitro* studies (Walsh et al., 2000) and molecular dynamics simulations (Manchester and Ornstein, 1995) showed that a smaller active site pocket promotes faster dehalogenation by increasing the likelihood of collisions between the halogen and the haem group. Substrate binding can be enhanced by increasing the hydrophobicity of the active site cavity or weakened by increasing its polarity (Walsh et al., 2000). However, making the active site more hydrophobic to enhance substrate binding also decreased the reaction rate. It is not clear whether this is due to increased retention of the hydrophobic product, sub-optimal substrate binding conformations, or decreased polarisation of the substrate. Increasing the hydrophilicity of the active site to weaken substrate binding did not substantially affect the reaction rate.

Rat P450 1A2 can also partially dechlorinate hexachloroethane to penta-, tetra-, and tri-chloro alkanes and alkenes (Yanagita et al., 1997), and studies of P450 1A2 mutants have yielded some important insights into reductive P450 metabolism. Most P450 enzymes have a conserved Asp/Glu-Thr motif on the distal side of the active site, where a single acidic residue is followed by a cluster of threonine residues. Experiments with mutants of rat P450 1A2 revealed that these threonine residues are important for oxygen binding and activation (Ishigooka et al., 1992). Some mutations decreased the rate of oxygen consumption to less than a third of the wild-type without disrupting the overall fold of the enzyme (Ishigooka et al., 1992). A Thr319Ala mutant of P450 1A2 can reduce hexachloroethane to tetrachloroethylene under aerobic conditions and perform reductive dechlorinations 2–10 times faster than the wild type under both aerobic and anaerobic conditions (Yanagita et al., 1997, 1998).

### Reductive Dechlorination of Carbon Tetrachloride

**FIGURE 4:**
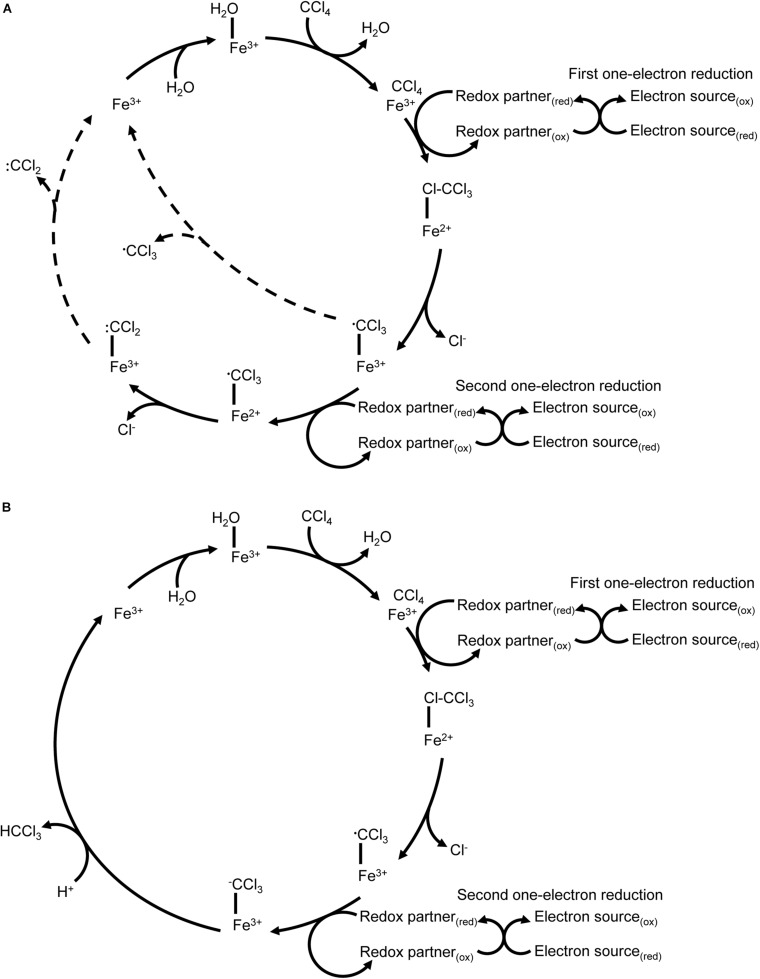
Reductive dechlorination of carbon tetrachloride. **(A)** P450 2E1 catalyses a one-electron reduction of carbon tetrachloride that results in release of chloride and formation of a carbon trichloride radical. The reaction cycle may uncouple with release of the carbon trichloride radical. A second one-electron reduction without protonation has also been reported, resulting in a carbon dichloride radical. Uncoupling events with release of radicals are indicated with dashed lines. **(B)** P450_*C*__*AM*_ catalyses a two-electron reduction of carbon tetrachloride to yield carbon trichloride.

In the twentieth century, carbon tetrachloride (CCl_4_) was a common industrial chemical used in dry cleaning solvents, fire suppressants and refrigerants (Doherty, 2000), among other chemical products. Its reductive dehalogenation was first identified in mammalian liver microsomes where its partial metabolism is associated with organ damage (McGregor, 1996). The first step in P450-mediated metabolism of carbon tetrachloride is a one-electron reduction by P450 2E1 (Zangar et al., 2000), resulting in a carbon trichloride radical and the release of a chloride ion. There are multiple potential fates for the radical depending on the reaction environment. Lipid peroxidation and inactivation of the P450 by radical products are possible outcomes, as is oxidation by molecular oxygen to form carbon dichloride and ultimately carbon dioxide. Further one-electron reductions of carbon trichloride result in a carbonyl dichloride radical that can also inactivate the P450 directly or be hydrolysed to carbon monoxide (a P450 inhibitor) (Li et al., 2014) (Figure 4A). This reaction cascade makes mammalian P450-mediated catabolism of carbon tetrachloride an interesting case study: by using reductive metabolism in hypoxic conditions the P450 can metabolise a toxic compound that cannot be metabolised via conventional oxidative P450 metabolism, but the products of reductive metabolism in this case pose a high risk of enzyme inactivation. Most mammalian carbon tetrachloride metabolism studies have focused on the release of radical intermediates due to their potential role in organ damage. By comparison, P450_*C*__*AM*_ catalyses a two-electron reduction of carbon tetrachloride to yield chloroform (CHCl_3_) without the release of identifiable quantities of radical intermediates, but P450_*C*__*AM*_ does not further reduce the chloroform product (Castro et al., 1985; Li and Wackett, 1993) (Figure 4B).

Complete reductive dehalogenation of carbon tetrachloride was achieved using electrobiocatalysis mediated by an immobilised thermostable enzyme, P450 119 from *Sulfolobus solfataricus* (Blair et al., 2004). Electrocatalysis using immobilised P450 enzymes is an established technology where the catalytic haem iron is reduced directly by an electrode (Sadeghi et al., 2011), forgoing the need for proteinaceous redox mediators and creating the opportunity to regulate the supply of electrons in strongly electronegative conditions that cannot be matched by fully biological systems. Thermostable enzymes are generally preferred in bioelectrochemistry for their longevity and the opportunity for high temperature catalysis.

Immobilised on a graphite electrode, the haem of P450 119 was reduced to Fe(I) which then catalysed a two-electron reduction of carbon tetrachloride, resulting in reductive dechlorination without the release of radical intermediates (Blair et al., 2004). This system was able to repeatedly dechlorinate the resulting products, ultimately releasing methane. The non-biological reduction potentials attainable with *ex vivo* electrobiocatalysis may present a route to complete dehalogenation of other substrates.

### Partial Dechlorination of Dichlorodiphenyltrichloroethane (DDT)

DDT is a potent insecticide that was used around the world in the mid-twentieth century before gaining notoriety for environmental harm. Its use was eventually banned in most countries. DDT accumulates in the food chain by concentrating in lipids and it has a wide range of adverse health effects on animals (Vieira et al., 2001). Under anaerobic conditions several P450 enzymes can catalyse a singular reductive dechlorination of DDT to produce dichlorodiphenyldichloroethane (DDD). This reaction has been identified with rat liver microsomes, several recombinant human P450s (primarily P450s 3A4, 2B6, 2E1, and 2C9) (Kitamura et al., 2002), and P450 6G1 from *Drosophila melanogaster* (Joußen et al., 2008).

However, this single dechlorination of the trichloromethane group does not appear to be a particularly challenging biochemical reaction. In anaerobic conditions DDT can also be reduced to DDD by incubation with NADH, FMN, and haemoglobin (Kitamura et al., 1999). The reaction is not enzymatic as such, because haem proteins retain this catalytic ability after denaturation by boiling. The product, DDD, is also highly toxic and recalcitrant in the environment (Mansouri et al., 2017) with limited evidence of further biotic dechlorination of this compound.

### Denitration and Bioremediation of Royal Demolition Explosive (RDX)

Reductive denitration reactions have also been identified in the P450 catalytic repertoire. As with reductive dehalogenations, these reactions were first observed in mammalian liver microsomes under anaerobic conditions. For example, human liver P450s from the CYP3A subfamily can denitrate glyceryl trinitrate (nitroglycerin), releasing nitric oxide and a mixture of di- and mono-nitrated products (Servent et al., 1989; Delaforge et al., 1993). The reaction cycle is similar to reductive dehalogenations as it proceeds via a P450 Fe(II)-NO intermediate, requires NADPH, and this reaction is competitively inhibited by oxygen.

P450-mediated reductive denitrations have become an important resource for bioremediation of nitrated explosives. RDX (or Royal Demolition Explosive) is the common name for 1,3,5-trinitro-1,3,5-triazinane, an explosive compound developed in the late nineteenth century. RDX has been used pervasively in military explosives and in civilian applications (such as demolition) up to the present day. Consequently, soil contamination with RDX is widespread (Jenkins et al., 2006) and of concern due to its toxicological profile (Burdette et al., 1988).

*Rhodococcus rhodochrous* strain 11Y, a bacterium isolated from explosive-contaminated soil, can utilise RDX as its sole nitrogen source. This strain harbours a P450 enzyme (XplA) that denitrates RDX and its intermediates and a reductase partner protein (XplB) (Seth-Smith et al., 2002). XplA catalyses the reductive denitration of RDX, and the reaction is likely the result of sequential one-electron reductions of the substrate and its subsequent products (Bhushan et al., 2003; Sabbadin et al., 2009). The enzyme is unusual in that it performs reductive denitrations under anaerobic or aerobic conditions, producing either methylenedinitramine plus one mole of nitrite and two moles of formaldehyde per mole of substrate (anaerobically), or 4-nitro-2,4-diazabutanal plus two moles of nitrite and one of formaldehyde (aerobically) (Sabbadin et al., 2009).

XplA isoforms have subsequently been discovered in soil microbes across the globe (Rylott et al., 2006; Halasz et al., 2012; Sabir et al., 2017) and its structural arrangement is unusual among characterised P450s. It has a flavodoxin domain fused to its N-terminus and it appears to have lost the conserved Asp/Glu-Thr motif from the active site cavity (Sabbadin et al., 2009). The Asp/Glu-Thr motif is important for oxygen binding and activation (Vidakovic et al., 1998; Nagano and Poulos, 2005) and this mutation appears to be essential for denitration in aerobic conditions. This is the same Asp/Glu-Thr motif that was mutated in P450 1A2 to enable reductive reactions in aerobic conditions (Yanagita et al., 1998) (described above in *Reductive dechlorination of halomethanes and haloethanes*). Reductive denitration of RDX is also catalysed by P450 2B4 from rabbit liver but the reaction is approximately three times faster in anaerobic conditions, suggesting that oxygen limits binding of RDX to the active site haem in P450 2B4 (Bhushan et al., 2003).

The opportunity to develop XplA for explosive bioremediation was recognised early. Expression of XplA in *Arabidopsis thaliana* enabled phytoremediation of contaminated soil. XplA decreased the concentration of RDX in aerial tissues and enhanced plant growth in soil containing what would ordinarily be inhibitory concentrations of RDX (Rylott et al., 2006). RDX removal from soil was up to 30 times faster when XplB, the cognate flavodoxin reductase, was coexpressed with XplA (Jackson et al., 2007).

Subsequent studies have worked toward RDX bioremediation solutions that could be deployed at contaminated sites. XplA overexpression was engineered in *Pseudomonas fluorescens*, resulting in a bacterium that can colonise plant roots and degrade RDX in the rhizosphere (Lorenz et al., 2013). However, experimental data indicated that haem biosynthesis in this bacterial strain limits P450 activity. Supplementation with aminolevulinic acid (a haem precursor) was necessary to enhance degradation of RDX in soil samples.

Several grass species overexpressing XplA and XplB have been engineered for bioremediation of former munitions ranges (Zhang et al., 2017, 2019). Grasses are desirable species for soil remediation due to their dense root systems and year-round ground cover (Zhang et al., 2017). Because of the prevalence of mixed explosive residues at actual contaminated sites, these plants were also engineered to coexpress a bacterial nitroreductase (NfsI) that can detoxify 2,4,6-trinitrotoluene (TNT) when overexpressed in plants (Bryant and DeLuca, 1991; Rylott et al., 2011). Engineered Western wheatgrass (Zhang et al., 2019), creeping bentgrass and switchgrass (Zhang et al., 2017) all metabolised RDX in liquid culture, and the engineered switchgrass also eliminated 15 mg of RDX from a 1.5 L column of gravel and sand.

## Concepts and Opportunities for Reductive Bioremediation With P450s

Reductive chemistry is initially important for dehalogenating compounds that cannot be enzymatically oxidised, but as halides are progressively eliminated and electron pairs become available for forming carbon-carbon double bonds, the products can become inert to further reduction. An example of this is the reductive metabolism of hexa- and penta-chloroethane described earlier in this review. Reaction sequences that result in 1,1,2,2-tetrachloroethane cannot be further dechlorinated via reductive reactions. 1,1,1,2-tetrachloroethane can be reductively dechlorinated to dichloroethylene but not further. Complete bioremediation of perhalogenated alkanes usually requires a combination of reductive and oxidative reactions. Compounds with fewer halide groups can often be oxidatively dehalogenated by a range of mono- and dioxygenases, and many perhalogenated alkanes could potentially be fully dehalogenated by a sequential cascade of reductive and then oxidative reactions.

**FIGURE 5:**
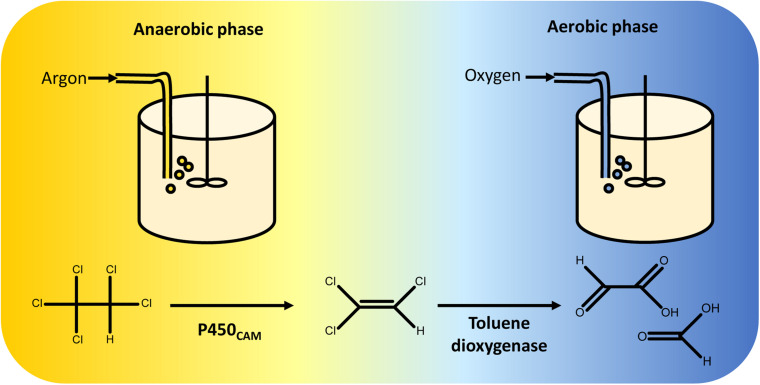
Two-stage fermentation for complete degradation of pentachloroethane. *Pseudomonas putida* was engineered to overexpress P450_*C*__*AM*_ and toluene dioxygenase. Anaerobic conditions were maintained during the first stage of the fermentation, enabling reductive dechlorination of pentachloroethane to 1,1,2-trichloroethylene by P450_*C*__*AM*_. The gas feed was then switched from argon to oxygen, facilitating oxidative metabolism of 1,1,2-trichloroethylene by toluene dioxygenase (Wackett et al., 1994). Toluene dioxygenase oxidises 1,1,2-trichloroethylene to an unstable 1,2-dihydroxytrichloroethane intermediate that decomposes into a mixture of glyoxylate and formate (Shuying and Wackett, 1992), both of which can be incorporated into *P. putida* central carbon metabolism.

Sequential reductive and oxidative reactions were used in one of the first synthetic biological pathways for complete catabolism of organohalides. In this example, P450_*C*__*AM*_ and a toluene dioxygenase (TOD) were co-expressed in *Pseudomonas putida* G786 (Wackett et al., 1994) to metabolise pentachloroethane. When the engineered strain is grown under anaerobic conditions, P450_*C*__*AM*_ reduces pentachloroethane to trichloroethylene. In the second stage of the fermentation, oxygen is introduced and TOD oxidises trichloroethylene to dihydroxytrichloroethylene. This product is unstable and decomposes to a mixture of glyoxylate and formate (Shuying and Wackett, 1992) (Figure 5).

This paradigm of P450-mediated reductive dehalogenation followed by TOD-mediated oxidation has been repeated elsewhere: the same engineered *P. putida* was used to metabolise tetrabromoethane and several and chlorofluoroethanes (Hur et al., 1994), and the concept was also successfully transferred to another host microbe. A P450_*C*__*AM*_ mutant (F87W, which is predicted to have enhanced pentachloroethane turnover kinetics (Manchester and Ornstein, 1995)) was functionally expressed in *Alcaligenes* sp. KF711 along with the a TOD that was fused to a native *Alcaligenes* ferredoxin and ferredoxin reductase (Iwakiri et al., 2004).

There are several potential advantages to using P450s to catalyse initial reductive dehalogenation steps in an engineered microbe instead of using reductive dehalogenases from organohalide respiring bacteria. P450s are naturally aerotolerant, whereas reductive dehalogenases are highly oxygen sensitive. The use of aerotolerant enzymes simplifies bacterial strain maintenance and fermentation procedures, particularly when designing a process that needs to switch between anoxic and aerobic conditions.

Studies of P450 1A2 (Yanagita et al., 1997, 1998) and the RDX-denitrifying P450 XplA (Sabbadin et al., 2009) indicate that mutating the Asp/Glu-Thr active site motif can activate reductive metabolism even in aerobic conditions by impairing oxygen binding and activation. P450 mutants that perform reductive reactions in aerobic conditions would be beneficial for synthetic bioremediation because of the superior energy yield of bacterial growth during aerobic respiration, which supports protein overexpression, high growth rates, and NAD(P)H supply. It remains to be seen whether these mutations can be transposed to other P450s as a general strategy for enabling reductive biochemistry under aerobic conditions.

The exceptional substrate promiscuity of mammalian P450s makes them a good starting point for identifying useful reactions. Even though reductive P450-mediated denitrations and dehalogenations were first discovered in mammalian systems, mammalian P450s are yet to be applied in synthetic bioremediation. Part of the reason may be the high rate of uncoupling observed in mammalian P450s after the first one-electron reduction, where damaging radical intermediates are released. However, this may not be generally representative of mammalian P450s. In some cases it appears that reductive dehalogenations of pharmaceuticals were only identified because of radical-associated organ damage (Minoda and Kharasch, 2001), biasing observations toward reactions that frequently uncouple and release radical products. Coupling efficiency can also be improved through mutagenesis (Jung et al., 2011), and given the vast landscape of P450 sequences it is conceivable that useful enzymes may be found for a greater range of reductive reactions than has been explored to-date.

There are even examples of substituting the P450 axial cysteine haem ligand to enhance specific reductive reactions. The axial cysteine was exchanged for serine in an engineered mutant of P450_*B*__*M*__3_ to increase the rate of reductive carbene transfer reactions in *E. coli* (Coelho et al., 2013). In this example the primary reason for the mutation was to increase the redox potential of the resting state low-spin haem iron, enabling *in vivo* reduction of the low-spin iron by NAD(P)H. This mutant was dubbed “P411” in recognition of the shift in the peak absorbance of the CO-bound ferrous haem, and it was used to catalyse reductive carbene transfers from ethyl diazoacetate to styrene. Similar mutations of the axial haem ligand may be useful for tuning the range of *in vivo* reductive dehalogenation reactions.

Cysteine ligation of the haem is also important for formation of the ferryl-porphyrin cation radical that catalyses the final monooxygenation step in the conventional P450 catalytic cycle. As such, mutation of the axial cysteine almost eliminated the formation of styrene oxide by P411 in aerobic conditions. However, it is important to note this mutation does not prevent futile oxidation of NAD(P)H to hydrogen peroxide (Vatsis et al., 2002), and reductive reactions of the P411 mutant are most productive under anaerobic conditions.

## New Frontiers and Future Directions in Synthetic Biology for Bioremediation

A classic example of using biocatalysts to remediate chemical pollution is the direct application of enzymes to a contaminated sample or site. For example, free atrazine chlorohydrolase enzyme can dechlorinate the herbicide atrazine in contaminated water (Scott et al., 2010). This relatively stable enzyme can be produced in bulk and applied directly to a sample because it does not require additional cofactors. This approach is not possible with P450s due to their requirement for reducing equivalents. Therefore, most P450-based bioremediation efforts focus on engineering their expression in whole organisms like plants or bacteria.

Plants are attractive host organisms for open-field bioremediation where low concentrations of chemicals need to be extracted from soil, especially when the pollutant is spread over a large area. A key concern in this scenario is containment of genetically modified organisms and their removal at the end of the bioremediation campaign. Designing biocontainment strategies for plants is an active field of research in the synthetic biology era with several promising approaches in development. These include engineering synthetic auxotrophies to limit the spread of plants, the development of sterile plants, and inducible transgene removal techniques (reviewed elsewhere; Clark and Maselko, 2020).

In published examples of plants engineered to remediate RDX, strong constitutive promoters are used to drive systemic P450 overexpression throughout the plant (Zhang et al., 2017, 2019). Systemic transgene overexpression frequently leads to gene silencing in engineered plants, especially when non-native promoters are used (Weinhold et al., 2013; Matsunaga et al., 2019). Organ- or even organelle-specific expression of P450s can improve expression stability (Bandopadhyay et al., 2010; Gnanasekaran et al., 2016) and creates the opportunity to be more selective about the reaction environment (Gnanasekaran et al., 2016; Mellor et al., 2018). For example expression in chloroplasts can be used to couple P450 activity to electrons generated by photosynthesis (Gnanasekaran et al., 2016).

Some pollutants accumulate in different plant tissues based on their chemical properties. For example, perfluoroalkyl substances (PFAS) have differential accumulation in plant tissues, with larger more hydrophobic compounds likely to accumulate in roots (Navarro et al., 2017). So targeted organ-specific expression of enzymes could be more efficient on the basis that enzyme expression can be directed to the region of the plant with the highest local concentration of substrate. If transport proteins for specific pollutant compounds can be identified, it may be possible to also direct the accumulation of pollutants into specific tissues or organelles. For example, transmembrane transport proteins linked to RDX transport have been identified (Rao et al., 2009). It may be possible to engineer their overexpression or knockout in a tissue-specific manner to specifically direct pollutants into sink tissues. This approach would be best suited to compounds that are recognised by reasonably specific transporters, as overexpression or repression of transporters important for plant homeostasis could be deleterious.

PFAS compounds are suspected to be taken up by plants via aquaporins, which are non-specific neutral solute transport channels (Mudumbi et al., 2017). Although these are non-specific channels required for water transport it may still be possible to enhance catabolism of aquaporin-transported pollutants. Synthetic protein scaffolding refers to actively co-localising different proteins through engineered protein-protein interactions (Behrendorff et al., 2020) and studies have demonstrated that some P450 enzymes can be scaffolded to membrane proteins (Henriques de Jesus et al., 2017; Behrendorff et al., 2019). Scaffolding P450s to relevant transmembrane transport proteins may be another way to enhance metabolism of compounds that are otherwise present at low concentrations by increasing the likelihood that a substrate will interact with the P450 enzyme by merit of physical proximity between the P450 and the transport channel. The advantages of using scaffolding to co-localise enzymes and transport channels have been reviewed previously (Behrendorff et al., 2020).

Bioremediation of contained liquid samples is conceptually less complex than remediation of contaminated environments in the field. Remediation of contained liquids allows for a high degree of process control and engineered bacteria can be employed using established microbial fermentation technologies. These advantages can also be extended to field applications when dealing with contaminated liquids that can be diverted into a contained system, such as extractable groundwater (Janssen and Stucki, 2020). New genetic tools for engineering different bacteria are reported regularly, and as such there are increasing options to engineer reductive P450 metabolism into bacteria that can already perform downstream steps in a bioremediation metabolic pathway. There are also increasing options to work with microorganisms that have other desirable properties like solvent tolerance (Sardessai and Bhosle, 2004) or thermotolerance (Shaw et al., 2008).

Difficulties in optimising bacterial expression of non-native P450s has previously been a hurdle to the development of microbial biocatalysts based on plant or mammalian P450s but recent studies indicate that these problems can be largely overcome by engineering the enzymes for increased folding stability (Gumulya et al., 2018; Tavanti et al., 2018). The proliferation of reliable methods for increasing the thermostability of P450s will dramatically expand the suite of reactions available for creating synthetic bioremediation reagents in plants, microorganisms, and perhaps even *ex vivo* technologies like bioelectrocatalysis or other immobilised enzyme systems where auxiliary enzymes like phosphite dehydrogenase regenerate the required reducing cofactors (Tan et al., 2015). Protein-based water filtration devices based on aquaporins (Xie et al., 2013) have been developed to commercial availability^2^. Combining a bio-based filtration system with the synthetic biology tools described above could lead to the development of water filtration systems that can enzymatically remove halogenated pollutants from a flowing water supply.

The use of reductive P450 metabolism in bioremediation has barely begun to be explored. New P450 sequences can be identified from bioinformatic databases (Nelson, 2018), and although enzyme function cannot yet be inferred from sequence, there is sufficient evidence that in general the P450 catalytic haem can reduce some substrates in the absence of oxygen. With a rapidly growing track record of engineering P450s for efficiency, stability, and selectivity, it appears that the opportunities to improve and deploy P450s as reductive biocatalysts for bioremediation are vast in the synthetic biology era.

## Author Contributions

JB conceived of and composed this manuscript.

## Conflict of Interest

The author declares that the research was conducted in the absence of any commercial or financial relationships that could be construed as a potential conflict of interest.

## Footnotes

1 http://rcsb.org

2 http://aquaporin.com
